# Dehydroepiandrosterone improves follicular fluid bone morphogenetic protein-15 and accumulated embryo score of infertility patients with diminished ovarian reserve undergoing in vitro fertilization: a randomized controlled trial

**DOI:** 10.1186/s13048-014-0093-3

**Published:** 2014-10-21

**Authors:** Huan H Zhang, Ping Y Xu, Juan Wu, Wei W Zou, Xia M Xu, Xia Y Cao, Lian Z Wei

**Affiliations:** Department of Reproductive Endocrinology, the Reproductive Medicine Center, The Affiliated Hospital of Anhui Medical University, Anhui, People’s Republic of China; Laboratory of the Reproductive Medicine Center, The Affiliated Hospital of Anhui Medical University, Anhui, People’s Republic of China

**Keywords:** Dehydroepiandrosterone, Diminished ovarian reserve, BMP-15, GDF-9, AMH

## Abstract

**Objective:**

To evaluate the effect of dehydroepiandrosterone (DHEA) on infertility patients with diminished ovarian reserve undergoing in vitro fertilization.

**Methods:**

This is a prospective study. Ninety-five patients with diminished ovarian reserve were included in this study. Of them, 42 patients were randomly allocated to the DHEA group, who received DHEA 75 mg daily for three consecutive menstrual cycles prior to IVF cycles, and 53 patients were allocated to the control group, who entered IVF cycles directly. All patients were treated with the same ovarian stimulation protocol. Follicular fluid samples from both groups were collected for bone morphogenetic protein-15 (BMP-15) and growth differentiation factor-9 (GDF-9). Fluid from the first aspirated follicle without any visible blood contamination was carefully collected. In addition, day 3 Blood samples were collected pre- and post-treatment of DHEA for serum anti-Mullerian hormone (AMH), follicle stimulating hormone (FSH) and estradiol (E_2_) in the DHEA group.

**Results:**

The level of BMP-15 in follicular fluid samples from the DHEA group was significantly higher than that of the control samples (*P=*.000). Patients after DHEA treatment demonstrated a significantly higher level of AMH and a significantly lower level of FSH, E_2_ compared to themselves prior to DHEA therapy (*P=*.015; *P=*.036; *P=*.002; respectively). Moreover, the accumulated score of embryos was significantly higher in the DHEA group (*P=*.033).

**Conclusions:**

These observations confirm the beneficial effect of DHEA for infertility patients with diminished ovarian reserve.

**Trial registration:**

ChiCTR-TRC-14005002

With the changing lifestyle including the increase pace of social life and delayed age of reproductive today, it is common to find infertility patients with Diminished ovarian reserve (DOR). DOR is a progressive decline of ovarian oocyte quality and quantity, with an elevated basal follicle stimulating hormone (FSH) level and less antral follicle count. It is reported in 9–24% of in-vitro fertilization (IVF) cycles [1]. Patients with DOR have high cancellation rate, low pregnancy rate, and high abortion rate during IVF [2].

Presently Dehydroepiandrosterone (DHEA) is utilized to improve the outcome of IVF cycles in about one third of reproductive centers around the world for accumulated surveys have shown the beneficial effect of DHEA on ovarian reserve. Casson *et al.* [3] observed the increase level of IGF-1 after DHEA supplementation. Study by Barad *et al.* [4] noted significant increases in oocyte, embryo numbers and better embryo grades after DHEA treatment through paired analysis of before and after IVF parameters of twenty-five patients with DOR. Subsequently, Barad *et al.* [5] got a conclusion that addition of DHEA gave rise to pregnancy rate meanwhile other reported a significantly reduction in Miscarriage rate [6].

However, the early reports on DHEA supplement to the patients with DOR were still controversial because there was lack of large-scale, well-designed confirmatory studies [4,7,8]. To further evaluate the effect of DHEA, ovarian reserve markers such as AMH, inhibin-B and antral follicle count were detected and significant changes were observed by N. Yilmaz *et al.* [9]. However, a small-sized randomized double-blinded placebo-controlled trial observed no significant changes in serum AMH and FSH levels [10].

Given this, studies should be continued to assess the utilization of DHEA in patients with DOR undergoing IVF. There are still no ideal markers predicting the quality of oocytes and embryos though there are numerous markers predicting response to ovarian induction. To our knowledge, follicular fluid contains many vital cytokines in reaction of oocyte quality. BMP-15, GDF-9, members of the transforming growth factor-β super family, are known to be of great importance for follicle growth and female fertility [11,12]. GDF-9 deficient female mice were blocked at primary one-layer follicle stage during follicular development which eventually leads to complete infertility [13]. High BMP-15 level in follicular fluid has been demonstrated to associate with high quality oocyte, high cleavage rate and good morphology embryos [14].

Therefore, to gain further insights on the effect of DHEA, here in our study, BMP-15, GDF-9 in follicular fluid were studied to obtain more evidence. The specific aims of the present study were: 1) To explore the levels of BMP-15 and GDF-9 in follicular fluid and compare them between the DHEA group and the control; 2) To confirm whether there are significant changes in ovarian reserve markers such as AMH, FSH, E2 after DHEA supplementation in the study group; 3) To compare the outcome of IVF cycles between the two groups.

## Materials and methods

### Patients

The study recruited patients with primary or secondary infertility for diminished ovarian reserve at the Assisted Reproductive Center, The Affiliated Hospital of Anhui Medical University between March 2013 and May 2014. Despite there is no established definition of DOR, predictive criteria such as abnormal ovarian reserve tests (day 3 FSH or AMH level) and previous ovarian response number of oocytes retrieved after a standard-dose ovarian stimulation are two of the most frequent criteria adopted on the topic today [15]. In this study the criteria for defining DOR was defined as follow: 1) an elevated day 3 FSH level ≥ 10 mIU/mL or FSH/LH > 3; 2) the number of antral follicle count less than five; 3) a previous poor ovarian response: retrieval of fewer than five oocytes or cycle cancellation due to poor response to ovarian stimulation. A diagnosed with DOR was reached if they fulfill any of the above three. Patients were excluded if they had any of the following: 1) a history of ovarian cystectomy or oophorectomy; 2) a diagnosis of endometriosis; 3) a history of DHEA supplementation or hormonal replacement therapy.

### Methods

This prospective study was approved by the Clinical Research Ethics Committee of The First Affiliated Hospital of Anhui Medical University. Before the trial, every participant was fully explained about the present study. A total of 105 patients fulfilled the selection criteria were recruited in our present study. According to a computer-generated randomization list by a nurse, 52 patients were randomly allocated to the DHEA group who received DHEA 25 mg three times per day for three consecutive menstrual cycles prior to IVF cycles and 53 patients to the control group who entered IVF cycles directly. On the first menstrual cycle and the fourth menstrual cycle, day 3 blood samples were collected for serum AMH, FSH, E2 in the DHEA group.

Everyone were given a dose of 225 IU human menopausal gonadotropin (HMG, Lizhu, China) combined with Clomiphene Citrate (CC, Lizhu, China) 100 mg daily from day 3 of the cycle to induce follicle growth. When at least one mature follicle of ≥ 18 mm in mean diameter was seen on ultrasound, a dose of 10,000 IU of Human chorionic gonadotropin (HCG, Lizhu, China) was administrated followed by oocyte retrieval 36 hour later.

Clean follicular fluid was aspirated from leading follicle by trans-vaginal ultrasound-guided puncture for BMP-15 and GDF-9 detection. Fluid samples from the first aspirated follicle and without any visible blood contamination were carefully collected. Samples were centrifuged at 2000 r/min for 15 minutes and stored at −80°C until to be tested.

### Outcome measures

The primary outcome measures were follicular fluid BMP-15, GDF-9 and serum AMH, FSH, E_2_. Secondary outcome measures were the number of oocytes retrieved, MII oocytes, and embryos transferred; the accumulated score of embryos.

### Sample detection

The concentration of BMP-15, GDF-9, AMH were determined by enzyme-linked immunosorbent assay (ELISA) [BMP-15: BMP-15 Gen II ELISA, USCN, USA; GDF-9: GDF-9 Gen II ELISA, USCN, USA; AMH: AMH Gen II ELISA, ASNL, USA]. FSH, E2 in serum samples were determined by a radioimmunoassay kit (Beckman Coulter).

### Embryo grade

On the third day of incubation, embryos were graded from one to four, based on percent fragmentation and cell counts: grade 4, equal-sized symmetrical cells with no fragmentation; grade 3, equal-sized symmetrical cells with, 10% fragmentation; grade 2, non-symmetrical blastomeres with 10-50% fragmentation and grade 1, 50% fragmentation [16]. Up to three, best-quality embryos were transferred on day 3 or day 5. Accumulated embryo score was calculated by summing the score of each embryo produced by each patient on the third day of development. For example, a patient with a grade 4 embryo and two grade 3 embryos would be assigned a score of 10. Patients with cycle cancellation would be assigned a score of 0.

### Statistical analyses

SPSS for Windows, Standard version 17.0 was utilized for data analysis. Quantitative variables were presented as mean ± standard deviation (SD) and analyzed by student’s t-test. Qualitative variables were presented as proportions and analyzed by χ2 test. For all analysis, the significant was set at *P*<.05.

## Results

**Figure 1:**
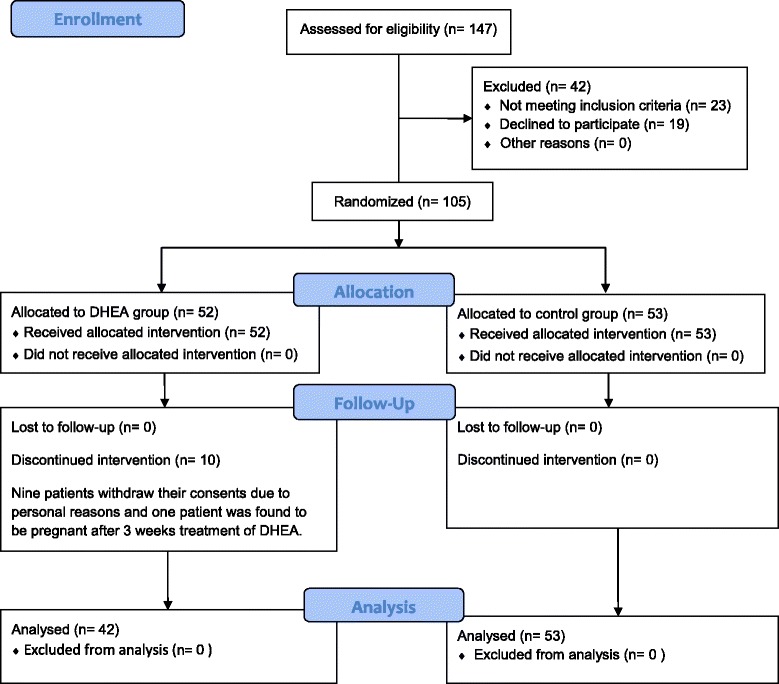
**Consort 2010 flow diagram.**

Between March 2013 and May 2014, a total of 147 patients with primary or secondary infertility for diminished ovarian reserve were screened at the Assisted Reproductive Center, The Affiliated Hospital of Anhui Medical University. Of them, 105 patients were recruited and randomized. Excluded 10 patients discontinued intervention; there were 42 patients in the DHEA group and 53 in the control. (See in Figure 1).

Patients enrolled in the DHEA group and the control group were aged 37.17 (±5.22) and 37.43 (±4.33) years respectively. As is shown in Table 1, all descriptive parameters were comparable between the DHEA group and the control group in terms of age, body mass index (BMI), duration of infertility, primary or secondary infertility (*P=*.785, *P=*.549, *P=*.756, *P=*.126 respectively). Moreover, serum AMH, FSH, E2, the total number of antral follicle were all matched in this two groups thus signifying they shared comparable ovarian reserve (*P=*.440; *P=*.421; *P=*.835; *P=*.764 respectively).

**Table 1:** **Basic characters of patients in the DHEA group and the control group**

**Paremeters**	**The DHEA group**	**The control group**	***P*** **-value**
	**n = 42**	**n = 53**	
Age (years)	37.17 ± 5.22	37.43 ± 4.33	.785
BMI (kg/m^2^)	22.13 ± 2.92	22.46 ± 2.42	.549
Duration of infertility(years)	6.64 ± 4.54	6.34 ± 4.85	.756
Primary infertility (%)	42.86 %	26.42%	.126
Secondary infertility (%)	57.14 %	73.58%	
AMH (ng/ml)	1.01 ± 0.77	1.12 ± 0.84	.440
FSH(mIU/mL)	11.68 ± 6.62	10.81 ± 2.41	.421
Estradiol (pg/ml)	186.58 ± 142.19	193.06 ± 156.16	.835
Antral follicle count	2.95 ± 1.38	3.04 ± 1.38	.764

Data are presented as mean ± SD (standard deviation) or percentage as appropriate. BMI: body mass index. *P*<.05 was considered as statistically significant.

**Figure 2:**
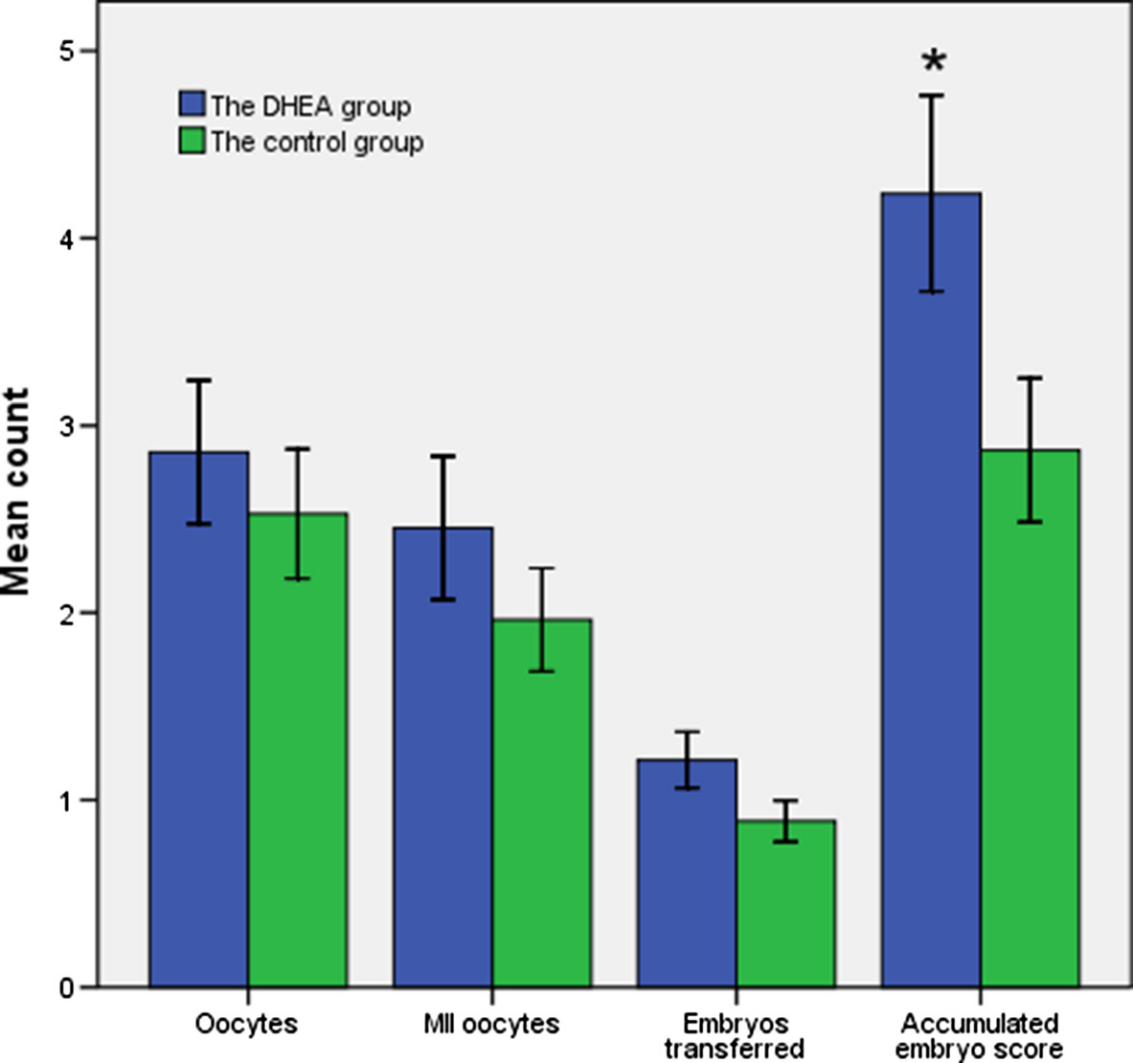
**Comparison of IVF cycle outcomes between the DHEA group and the control.**

Characteristics of IVF cycles regarding with the number of oocytes, MII oocytes, embryos transferred, and the score of embryos were also analyzed between the DHEA group (n = 42) and the control group (n = 53). As is shown in Figure 2, the accumulated score of embryos was significantly higher in patients treated with DHEA compared to the control group (4.24 ± 3.39 vs. 2.87 ± 2.79, *P=*.033). However, no significantly changes were observed in the number of oocytes retrieved, MII oocytes and embryos transferred (*P=*.526, *P=*.289, *P=*.076 respectively).

After standardized ovarian stimulation, 26 follicular fluid samples were collected from the DHEA group, with 6 cycles canceled for poor responses and 10 follicular fluid samples unqualified for visible blood contamination. In the control group, we sampled 35 samples with 9 cycles canceled and 9 unqualified respectively.

Basic characters of the DHEA follicular fluid (FF) group and the control follicular fluid (FF) group are presented in Table 2. No significant differences were found in terms of serum AMH, FSH, E2, and the total number of antral follicle between the two groups (*P=*.496; *P=*.406; *P=*653; *P=*.486 respectively). The mean levels of BMP-15 in the DHEA FF group (n = 26) and the control FF group (n = 35) were 0.81 (±0.25) ng/ml and 0.35 (±0.23) ng/ml respectively. And a statistically significant difference was found between the two FF groups (*P=*.000). No significant difference was detected in the level of GDF-9 in the DHEA FF group compared to the control (7.91 ± 3.77 vs. 6.82 ± 2.46, *P=*.203).

**Table 2:** **The outcome comparison between the DHEA FF group versus the control FF group**

**Paremeters**	**The DHEA FF group**	**The control FF group**	***P*** **-value**
	**n = 26**	**n = 35**	
Age (years)	36.58 ± 5.95	37.83 ± 4.28	.367
BMI (kg/m^2^)	22.49 ± 3.10	22.66 ± 2.39	.813
Duration of infertility(years)	7.04 ± 4.99	6.60 ± 5.01	.736
Primary infertility (%)	42.31%	25.71%	.270
Secondary infertility (%)	57.69%	74.29%	
AMH (ng/ml)	1.00 ± 0.81	1.19 ± 0.94	.496
FSH (mIU/mL)	11.78 ± 4.92	10.70 ± 2.40	.406
Estradiol (pg/ml)	174.47 ± 122.85	190.45 ± 145.87	.653
Antral follicle count	3.73 ± 1.04	3.51 ± 1.29	.486
BMP-15 (ng/ml)	0.81 ± 0.25	0.35 ± 0.23	.000
GDF-9 (ng/ml)	7.91 ± 3.77	6.82 ± 2.46	.203

Data are presented as mean ± SD (standard deviation) or percentage as appropriate. FF: Follicular fluid. *P*<.05 was considered as statistically significant.

A comparison of ovarian reserve markers was made between before and after treatment in the 42 patients treated with DHEA. The mean duration of DHEA supplementation was 83.67 ± 6.45 days. The levels of DHEA-S and testosterone were significantly higher after treatment (1.24 ± 0.74 μg/mL vs. 5.50 ± 3.48 μg/mL, p=.000; 0.74 ± 0.41 nmol/L vs. 2.25 ± 1.28 nmol/L, p=.000). And there was a significant increase of AMH (1.01 ± 0.77 ng/ml vs. 1.29 ± 1.09 ng/ml, P=.015) and a significant decrease of FSH (11.68 ± 6.62 IU/L vs. 9.45 ± 5.09 IU/L, *P=*.036) and E_2_ (186.58 ± 142.19 pg/ml vs. 110.79 ± 68.28 pg/ml, *P=*.002) after about 12 weeks of DHEA supplementation. However no significant change was found in the count of antral follicle (2.95 ± 1.38 vs. 3.21 ± 1.22; *P=*.054). (See in Table 3).

**Table 3:** **Ovarian reserve markers in patients before and after DHEA treatment**

**Paremeters**	**Pre-treatment** **n=42**	**Post-treatment** **n=42**	***P*** **-value**
AMH (ng/ml)	1.01 ± 0.77	1.29 ± 1.09	.015
FSH (mIU/mL)	11.68 ± 6.62	9.45 ± 5.09	.036
Estradiol (pg/ml)	186.58 ± 142.19	110.79 ± 68.28	.002
DHEA-S (μg/mL)	1.24 ± 0.74	5.50 ± 3.48	.000
Testosterone(nmol/L)	0.74 ± 0.41	2.25 ± 1.28	.000
Antral follicle count	2.95 ± 1.38	3.21 ± 1.22	.054

Data are presented as mean ± SD (standard deviation). *P*<.05 was considered as statistically significant.

In these 42 patients received DHEA treatment, eight patients conceived after the IVF cycles, with a pregnancy rate of 19.05%. In the control, seven patients conceived with a pregnancy rate of 13.21%. And there was no significant difference in pregnancy rate between them (*P=*.816).

During this trial, no major adverse effects were reported. Only one patient complained of dizziness and three patients complained of acne.

## Discussion

This is the first report concerning the relationship between the DHEA supplementation for patients with DOR and the level of BMP-15 and GDF-9 in follicular fluid. The present study found a significantly higher level of BMP-15 in follicular fluid of patients treated with DHEA prior to IVF cycles than patients entered IVF cycles directly.

Human data demonstrated that high BMP-15 level in follicular fluid is associated with high quality oocyte and good morphology embryos [11]. Moreover, poor responders with a high level of BMP-15 in follicular fluid demonstrated higher implantation rate and pregnancy rate than those in the low BMP-15 group [17]. Here in our study, the higher level of BMP-15 observed in DHEA treated group predicted better ovarian reserve and outcome of IVF cycles.

Additionally, some other ovarian reserve markers such as serum AMH, FSH, E_2_ and the count of antral follicle were measured before and after the supplementation of DHEA in this article. There was a significant higher level of AMH, a significantly lower level of FSH and E2 after about 12 weeks treatment of DHEA. However, no significant change was observed in the count of antral follicle in our study. Research also noted that DHEA supplementation improved serum AMH, inhibin B and antral follicle count in patients with DOR. Serum level of AMH increases in parallel with length of DHEA supplementation [18]. Subsequently, other study observed a significant increase in AFC and ovarian volume but no significant differences were found in the levels of AMH and FSH [10].

Furthermore, main IVF cycle characteristics were compared between the two groups in our study. The accumulated score of embryos were significantly higher in patients treated with DHEA. Thus signified a better quality of embryos and strengthened the observations in ovarian reserve markers above. And there was a trend for increased number of oocytes retrieved, MII oocytes and embryos transferred in the presence of DHEA, though this effect was not statistically significant.

In agreement with our study, Barad *et al.* [4] reported that DHEA treatment gave rise to a significant increase in oocyte, embryo numbers and better embryo grades through paired analysis of before and after IVF parameters of twenty five patients with DOR. In a late follow up, the first prospectively randomized clinical trial was published which suggested that addition of DHEA increased embryo quality and live birth rates in patients with DOR [16]. However, this paper has not accepted well as for its low number of 33 subjects with 17 in the DHEA group and 16 in the control. Subsequently in 2012, a study enrolled 133 DOR patients with 67 used DHEA and 63 without DHEA was reported. It demonstrated that peak E_2_, endometrial thickness, number of oocytes retrieved, number of embryos transferred in the study group were significantly higher than the control [19].

All of these observations raise a question, what may probably be the mechanism by which DHEA exert its effect on ovarian reserve? Recent data mostly focus on androgens receptor (AR), hold the opinion that androgens primarily affect granulose cells (GC) with ligand-activated AR [20]. AR-knockout (ARKO) female mice model revealed low number of antral follicles, fewer ovulated oocytes, significantly higher rates of GC and follicles apoptosis, poor responses to ovarian induction with gonadotropins [21]. ARKO model demonstrated that ARs are crucial important for normal follicle genesis and development. Higher concentration of AR mRNA and AR protein were detected in pre-antral and early antral follicles which suggested the peak effect of androgens [22].

To gain further light into the effect of androgens, pre-ovulatory granulose cells in follicular fluid were collected from patients undergoing IVF treatment, and cultured with the absence or presence of a low concentration of DHEA-S by ElBeltagy [23]. Increased expression of AR was detected after treatment of DHEAS compared to cells absence of DHEAS. This study explained that DHEAS may exert its effect on follicle development through up-regulating AR.

Human GC and follicular fluid from small antral follicles were obtained from ovaries surgically removed for fertility preservation by Nielsen *et al.* [24], who noted the expression of AR mRNA in GC and concentrations of androgens in follicular fluid were correlated with the expression of FSH receptor mRNA. Moreover, the expression of AR mRNA also showed a positive association with AMH receptor in their research. This indicated that proper androgens may potentially increase FSH receptor and AMH receptor.

In one word, androgens appear most modulate FSH responsibility through AR at stages of pre-antral and antral follicles [25]. Addition of DHEA could increase FSH receptor at early stages of folliculogenesis. Therefore DHEA could beneficially affect follicle recruitment.

Additionally, the secondary effect of DHEA on estrogen receptor could not be neglected. According to the two cell/two gonadotrophin theory, androgens play an essential role in ensuring adequate follicular steroidogenesis in human. DHEA is a crucial precursor steroid to human sex steroid synthesis and is converted to androgens or estrogens. Estrogens promote ovarian follicular growth and granulosa cell proliferation [26].

DHEA promotes the growth of small follicles, especially preantral and early-antral follicles. Since AMH is secreted by them, it is not difficult to understand the increase of AMH. However, the increase of AFC did not reach statistically difference. Why? Pre-antral and early-antral follicles could not be seen on ultrasound. We can only monitor the count of antral follicles, not the total number of pre-antral , early-antral and antral follicles. And AMH may reflect not only the quantity but also the quality of small follicles. The increase of AMH may be caused by the increased small follicles or the improved follicle quality or the both.

Here in our study, a significantly higher level of BMP-15 in follicular fluid of patients treated with DHEA was also observed. We speculated that DHEA may probably affect the level of BMP-15 in circulation via androgen receptors (AR). It has been demonstrated that multiple regulatory factors such as tyrosine kinases by Ligand (KITL), BMP-15, GDF-9 and so on are under the regulation of AR [27]. And there exists an exchange loop between oocytes and granulose cells.

It has been confirmed that both GDF-9 and BMP-15 stimulate FSH receptor and increase the sensitivity to FSH but only BMP-15 was observed to up-regulate AMH expression at mRNA and protein level in GC [28]. What is more, recent research indicated that high level of FSH suppressed the expression of BMP-15, and there is a negatively correlation between them [29]. It may be inferred that here exist a feedback loop between BMP-15 and FSH. Therefore in present study, it is reasonable for us to assume that DHEA supplementation may potentially enhance FSH sensitivity and increase the expression of BMP-15 in follicular fluid. However, no significant difference was found in the level of GDF-9. It is likely that there exists some difference in the paths of regulating oocyte growth between BMP-15 and GDF-9 though they make function in a synergistic way. And the relationships between BMP-15 and androgens need further research to clarify.

### Strengths and limitations

Strengths of this study were: 1) It was the first study concerning the relationship between DHEA supplementation and BMP-15, GDF-9 in follicular fluid. 2) Follicular fluid samples were collected distinguished serum samples in former studies assessed the effect of DHEA; 3) This research was conducted in a prospective way which ensured the consistency and comparability of the study group and the control.

Limitations of the study: It is not a placebo-controlled trial for patients with DOR were eager to receive treatment other than take placebo pills because of limited time to fertility.

## Conclusions

In summary, DHEA supplementation improves follicular fluid BMP-15, serum AMH and accumulated embryo score of patients with DOR. Herein, addition of DHEA prior to IVF-cycles is an effective option for patients with DOR.

## Abbreviations

DHEA: Dehydroepiandrosterone
DOR: Diminished ovarian reserve
IVF: In vitro fertilization
BMP-15: Bone morphogenetic protein-15
GDF-9: Growth differentiation factor-9
AMH: Anti-Mullerian hormone
FSH: Follicle stimulating hormone
E_2_: Estradiol
